# Safety of switching to brexpiprazole in Japanese patients with schizophrenia: A post‐hoc analysis of a long‐term open‐label study

**DOI:** 10.1002/hup.2777

**Published:** 2021-01-26

**Authors:** Jun Ishigooka, Ken Inada, Kazunari Niidome, Kazuo Aoki, Yoshitsugu Kojima, Shuichi Iwashita, Sakiko Yamada

**Affiliations:** ^1^ Institute of CNS Pharmacology Tokyo Japan; ^2^ Department of Psychiatry Tokyo Women's Medical University School of Medicine Tokyo Japan; ^3^ Department of Medical Affairs Otsuka Pharmaceutical Co., Ltd. Tokyo Japan; ^4^ Headquarters of Clinical Development Otsuka Pharmaceutical Co., Ltd. Osaka Japan

**Keywords:** brexpiprazole, Japanese, switching, safety, schizophrenia

## Abstract

**Objectives:**

To determine the long‐term safety of switching to brexpiprazole from aripiprazole or non‐aripiprazole dopamine antagonists.

**Methods:**

Post‐hoc analysis of 56‐week study of Japanese outpatients with schizophrenia switched to brexpiprazole 2 mg/day over 4‐week switching period with further titration (1–4 mg/day) allowed during the 52‐week, open‐label period. Major assessment items: total/low‐density lipoprotein (LDL)‐/high‐density lipoprotein (HDL)‐cholesterol, triglycerides, blood glucose, body weight and prolactin. Secondary evaluations were related to efficacy, treatment emergent adverse events (TEAEs), extrapyramidal symptoms, and corrected QT interval (QTc).

**Results:**

84/186 (45.2%) patients (aripiprazole, 32.9%; non‐aripiprazole, 54.8%) discontinued treatment over 56 weeks mainly because of consent withdrawal/adverse events. From baseline to Week 56, both groups showed minimal mean changes in total/LDL‐/HDL‐cholesterol, triglycerides, and glucose levels and a slight increase in mean (SD) body weight (aripiprazole, 1.1 [4.4] kg; non‐aripiprazole, 0.4 [4.6] kg). Mean prolactin levels increased slightly in the aripiprazole group, but decreased in the non‐aripiprazole group. Symptom severity scores decreased similarly in both groups. TEAEs occurred in 161/186 (86.6%) patients (aripiprazole, 84.1% [serious, 9.8%]; non‐aripiprazole, 88.5% [serious, 14.4%]). Few changes occurred in extrapyramidal symptom scales or QTc interval.

**Conclusions:**

Switching to brexpiprazole is associated with a low long‐term risk for metabolic abnormalities (including weight gain), hyperprolactinemia, extrapyramidal symptoms and QTc changes and minimal changes in psychiatric symptoms.

## 1 INTRODUCTION

Medication adherence is vital in the treatment of schizophrenia to ensure stable medication levels and prevent psychotic exacerbation and relapse. However, medication adherence is often poor among patients with schizophrenia because of attitudes to antipsychotics caused by present and past adverse events of antipsychotic drugs (Dibonaventura, Gabriel, Dupclay, Gupta, & Kim, 2012; Lambert et al., 2004; Velligan et al., 2009), as well as lack of insight related to the disease (Kalkan & Kavak Budak, 2020; Kim et al., 2019). Regarding adverse events, data from a survey of 876 adults with schizophrenia found that extrapyramidal symptoms/agitation, sedation/cognition, prolactin/endocrine effects and metabolic side effects, including weight gain, were all significantly associated with lower rates of antipsychotic medication adherence (Dibonaventura et al., 2012). Although antipsychotics should be chosen to reduce such adherence‐related issues, they are associated with many adverse events, particularly metabolic abnormalities, sexual dysfunction and extrapyramidal symptoms (Cooper et al., 2016; Cutler, 2003; de Boer, Castelein, Wiersma, Schoevers, & Knegtering, 2015; Hennekens, Hennekens, Hollar, & Casey, 2005; Inder & Castle, 2011; Newcomer et al., 2009; Olfson, Uttaro, Carson, & Tafesse, 2005; Taylor, Barnes, & Young, 2018). When adverse events occur in patients with schizophrenia, it may be necessary to consider switching to another antipsychotic. When switching, medication choice should take into account the adverse events profile of the pre‐switch and post‐switch agent and the tolerability concerns of individual patients (Buckley & Correll, 2008).

Brexpiprazole is a serotonin‐dopamine activity modulator that acts as a partial agonist at serotonin 5‐HT_1A_ and dopamine D_2_ receptors, and as an antagonist at serotonin 5‐HT_2A_ receptors (Maeda et al., 2014; McEvoy & Citrome, 2016). Brexpiprazole is approved in more than 40 countries for the treatment of schizophrenia and is also approved as an adjunctive therapy to antidepressants for the treatment of major depressive disorder in several countries, including the United States.

The results of placebo‐controlled short‐term and long‐term clinical trials from Japan and other countries have shown that brexpiprazole has a favorable safety profile and is generally well tolerated (Correll et al., 2015; Fleischhacker et al., 2017; Ishigooka, Iwashita, & Tadori, 2018a, 2018b; Kane et al., 2015). The most frequently noted adverse events include akathisia, headache, insomnia, and agitation although, in some studies, the incidence of these events was similar to or lower than that of placebo and most events were mild to moderate in severity (Correll et al., 2015; Fleischhacker et al., 2017; Ishigooka et al., 2018a, 2018b; Kane et al., 2015). The risk of weight gain, which is a known adverse event induced by atypical antipsychotics, has been noted to be lower with brexpiprazole than many other antipsychotics with weight gain of less than 2 kg observed in a short‐term study over 6 weeks (Correll et al., 2015; Kane et al., 2015).

Despite this favorable tolerability profile, there has been a relative lack of studies examining the safety and efficacy of switching to brexpiprazole. A post‐hoc analysis (Correll et al., 2019) of a 52‐week double‐blind, placebo‐controlled maintenance withdrawal study (Fleischhacker et al., 2017) found that discontinuation rates at 8 weeks were low and improvements in efficacy were similar in patients switched to brexpiprazole, regardless of the amount of time spent in the conversion phase, whereas adverse events were lower in those converted over a longer period (22–33 days). These results focus on the outcomes related to only 8 weeks' treatment, and there have been no results for the long‐term safety and efficacy of switching to brexpiprazole.

A recent long‐term open‐label extension study (Ishigooka, Iwashita, & Tadori, 2018b), conducted in Japan, examined the safety and effectiveness of brexpiprazole in both rollover patients who completed a short‐term, randomized, placebo‐controlled trial (Ishigooka et al., 2018a) and *de novo* patients who switched from other antipsychotics. Treatment‐emergent adverse events with brexpiprazole in Japanese patients with schizophrenia were similar to other trials (Correll et al., 2015; Fleischhacker et al., 2017; Forbes et al., 2018; Ishigooka et al, 2018a, 2018b; Kane et al., 2015) and most were mild or moderate in severity and there were no clinically significant changes in electrocardiogram parameters, bodyweight, laboratory values, or vital signs (Ishigooka et al., 2018b).

The aim of the present study was to determine the long‐term safety of switching to brexpiprazole in Japanese patients who entered the long‐term open‐label extension study and to explore the difference in safety between switching from aripiprazole (dopamine partial agonist) and non‐aripiprazole (dopamine antagonist) antipsychotics. This grouping and classification of agents was performed based on the known actions of these agents on the dopamine D_2_ receptor (Stahl et al., 2013).

## 2 METHODS

### 2.1 Study design

This study was a post‐hoc analysis of an open‐label, long‐term study of brexpiprazole administration in Japanese outpatients with schizophrenia (ClinicalTrials.gov NCT01456897; Ishigooka et al., 2018b). Data for this analysis were derived from de novo outpatients enrolled in the previous long‐term open‐label study, who were eligible if they were: aged ≥18 years, diagnosed with schizophrenia (DSM‐IV‐TR diagnostic criteria), receiving treatment with oral antipsychotics (other than clozapine), requiring chronic antipsychotic treatment per the study investigator opinion, and considered for monotherapy with brexpiprazole. Exclusion criteria and prohibited medications during the treatment period were published previously and are listed in Table S1. The present analysis covers the entire 56‐week evaluation period of the open‐label study, which comprised a (i) 4‐week switching period and (ii) a 52‐week open‐label brexpiprazole monotherapy period.

The open‐label study which formed the basis for this post‐hoc analysis was conducted in compliance with the ethical guidelines with protocols approved at each investigational site and written informed consent was obtained from all participating patients.

### 2.2 Treatment

Target patients for this post‐hoc analysis were de novo outpatients who entered the open‐label study and had received an atypical antipsychotic as the main agent. Based on the pretreatment atypical antipsychotic that was the main agent, patients were analyzed in groups divided into a dopamine partial agonist (aripiprazole) or a dopamine antagonist (non‐aripiprazole), including olanzapine, quetiapine, risperidone, paliperidone, blonanserin, and perospirone. In patients who had received more than one antipsychotic concomitantly, the main antipsychotic was defined as that with the highest chlorpromazine equivalent dose of antipsychotics 30 days before consent was obtained.

During the 4‐week switching period, patients were switched from their prior antipsychotic therapy to brexpiprazole 2 mg/day using an add‐on and taper‐off method. Specifically, brexpiprazole 1 mg/day was added to the previous antipsychotic regimen and the dose was increased to 2 mg/day after 2 to 3 weeks. The previous antipsychotic(s) were reduced from 2 weeks and discontinued after 4 weeks. During the subsequent 52‐week, open‐label period, patients were started at a dose of brexpiprazole 2 mg/day, and the dose was titrated, if necessary, within the range of 1 to 4 mg/day depending on Clinical Global Impression‐Improvement (CGI‐I) or tolerability issues.

### 2.3 Outcomes

Major assessment items included in this analysis were related to safety and consisted of metabolic parameters, including cholesterol levels (total cholesterol, low‐density lipoprotein (LDL)‐cholesterol, high‐density lipoprotein (HDL)‐cholesterol, triglycerides), and blood glucose levels; body weight; and serum prolactin levels. In addition, the following assessments were included for secondary evaluation: efficacy, as assessed by the Positive and Negative Syndrome Scale (PANSS) and the CGI‐Severity (CGI‐S); treatment emergent adverse events (TEAEs); extrapyramidal symptom rating scales, including the Abnormal Involuntary Movement Scale (AIMS), Barns Akathisia Rating Scale (BARS), and Drug‐induced Extrapyramidal Symptoms Scale (DIEPSS); and the corrected QT interval (QTc).

### 2.4 Statistics

Descriptive statistics related to mean change in quantity and frequency of values or patient numbers, respectively, were tabulated for patients who received brexpiprazole for 56 weeks (switching and open‐label periods) and results were divided into those who had previously received aripiprazole or non‐aripiprazole antipsychotics. This main classification was based on the action of aripiprazole and non‐aripiprazole antipsychotics on the dopamine D_2_ receptor. In addition, results for patients treated with non‐aripiprazole antipsychotics were further divided into those who had previously received risperidone or paliperidone (i.e., serotonin‐dopamine antagonists) and those who had previously received olanzapine or quetiapine (i.e., multi‐acting receptor‐targeted antipsychotics; Mauri et al., 2014). With regard to prolactin levels, values within the reference value range (men, 3.58–12.78 ng/ml; women, 6.12–30.54 ng/ml) were defined as normal. Values above and below these reference values were defined as high and low, respectively. In addition, the percentages of patients with low, normal, or high prolactin levels were tabulated at baseline, Week 4, Week 8, Week 28, and Week 56. With regard to body weight, the transition in average change in body weight according to baseline body mass index (BMI) groups (“underweight” [<18.5 kg/m^2^], “normal” [18.5–24.9 kg/m^2^], “overweight” [25–29.9 kg/m^2^], and “obese” [≥30 kg/m^2^]) was shown at baseline, Week 4, Week 8, Week 28, and Week 56. Overall, the change in the mean value of each item was calculated using observed cases. It should be noted that no formal statistical tests were carried out and only descriptive statistics were performed.

## 3 RESULTS

### 3.1 Subject disposition and baseline characteristics

Of a total of 250 de novo patients screened for eligibility for the open‐label study, 42 patients failed screening such that 208 patients were initially enrolled at the start of the 4‐week switching period and received brexpiprazole. Of these 208 treated patients, 22 patients were excluded due to pretreatment antipsychotics being a typical antipsychotic (*n* = 14), protocol deviation (*n* = 6), and antipsychotics not taken 30 days before the date of consent (*n* = 2), thus leaving a total analysis population of 186 patients for this post‐hoc analysis.

Over the 56‐week evaluation period, 84 of 186 (45.2%) patients discontinued treatment, including 27 of 82 (32.9%) patients in the aripiprazole group and 57 of 104 (54.8%) patients in the nonaripiprazole group. The most common reasons for discontinuation were withdrawal of consent (aripiprazole, 20.7% [17/82]; nonaripiprazole, 27.9% [29/104]) and adverse events (aripiprazole, 8.5% [7/82]; nonaripiprazole, 15.4% [16/104]). Exacerbation of schizophrenia was the most common adverse event that led to discontinuation (aripiprazole, 6.1% [5/82]; nonaripiprazole, 12.5% [13/104]). Overall, the mean daily dose of brexpiprazole at 56 weeks was 3.12 mg (aripiprazole, 3.11 mg; nonaripiprazole, 3.13 mg).

Demographic and baseline clinical characteristics of enrolled patients treated with brexpiprazole during the switching phase are shown in Table 1 (aripiprazole and nonaripiprazole) and Table S4 (aripiprazole, risperidone/paliperidone, olanzapine/quetiapine). Patients in the nonaripiprazole group were older, had longer duration of illness, and had a greater proportion of patients with concomitant use of antipsychotic drugs, benzodiazepines, anti‐parkinsonian drugs, and laxatives. Furthermore, baseline PANSS and CGI‐S scores were higher in the nonaripiprazole group compared with the aripiprazole group. Patients previously treated with nonaripiprazole antipsychotics also had a higher daily chlorpromazine equivalent dose at baseline (554.3 ± 421.4 mg/day for the nonaripiprazole group vs. 433.4 ± 258.8 mg/day for the aripiprazole group).

**TABLE 1:** Demographic and baseline clinical characteristics

	Aripiprazole (*n* = 82)	Non‐aripiprazole (*n* = 104)	Total (*n* = 186)
Age (years), mean ± SD	40.1 ± 12.9	49.1 ± 15.2	45.1 ± 14.9
Duration of illness (years), mean ± SD	12.1 ± 10.6	20.4 ± 15.3	16.7 ± 14.0
Female sex, *n* (%)	48 (58.5)	51 (49.0)	99 (53.2)
Body weight (kg), mean ± SD	66.5 ± 13.7	64.7 ± 14.8	65.5 ± 14.3
BMI (kg/m^2^), mean ± SD	24.9 ± 4.1	24.4 ± 4.7	24.6 ± 4.5
<18.5, *n* (%)	4 (4.9)	10 (9.6)	14 (7.5)
18.5 to < 25, *n* (%)	39 (47.6)	50 (48.1)	89 (47.8)
25 to < 30, *n* (%)	31 (37.8)	33 (31.7)	64 (34.4)
≥30, *n* (%)	8 (9.8)	11 (10.6)	19 (10.2)
CP equivalent dose of all antipsychotics (mg/day), mean ± SD	433.4 ± 258.8	554.3 ± 421.4	501.0 ± 363.1
Number of pretreatment antipsychotic drugs, *n* (%)			
1 drug	72 (87.8)	74 (71.2)	146 (78.5)
2 drugs	9 (11.0)	22 (21.2)	31 (16.7)
≥3 drugs	1 (1.2)	8 (7.7)	9 (4.8)
PANSS total score, mean ± SD	64.6 ± 19.1	74.6 ± 22.9	70.2 ± 21.8
CGI‐S, mean ± SD	3.2 ± 0.9	3.7 ± 1.2	3.4 ± 1.1
Concomitant medication use			
Benzodiazepines, *n* (%)	33 (40.2)	56 (53.8)	89 (47.8)
Non‐benzodiazepines, *n* (%)	1 (1.2)	2 (1.9)	3 (1.6)
Antiparkinsonian drugs, *n* (%)	8 (9.8)	23 (22.1)	31 (16.7)
Laxatives, *n* (%)	9 (11.0)	31 (29.8)	40 (21.5)

*Note*: Non‐aripiprazole breakdown: olanzapine (*n* = 36), risperidone (*n* = 33), paliperidone (*n* = 16), blonanserin (*n* = 8), quetiapine (*n* = 8), perospirone (*n* = 3).

Abbreviations: BMI, body mass index; CP, chlorpromazine; CGI‐S, Clinical Global Impression‐Severity; PANSS, Positive and Negative Symptom Severity; SD, standard deviation.

### 3.2 Major safety assessment items

In terms of metabolic parameters, mean changes in total cholesterol, triglycerides, LDL‐cholesterol, HDL‐cholesterol and glucose levels from baseline to Week 56 were small in both aripiprazole and nonaripiprazole groups (Table 2) and varied in aripiprazole, risperidone/paliperidone, and olanzapine/quetiapine groups (Table S5).

**TABLE 2:** Changes in lipid, blood glucose, and prolactin levels during 56‐week brexpiprazole treatment in patients previously treated with aripiprazole and nonaripiprazole antipsychotics (observed cases)

	Aripiprazole	Non‐aripiprazole	Total
Total cholesterol, mg/dl			
Baseline, mean ± SD (*n*)	191.7 ± 36.7 (82)	191.9 ± 37.3 (104)	191.8 ± 37.0 (186)
Change at Week 56, mean ± SD (*n*)	3.4 ± 23.9 (55)	9.5 ± 34.0 (47)	6.2 ± 29.0 (102)
Triglycerides, mg/dl			
Baseline, mean ± SD (*n*)	120.8 ± 111.5 (82)	128.5 ± 107.6 (104)	125.1 ± 109.1 (186)
Change at Week 56, mean ± SD (*n*)	−5.4 ± 79.9 (55)	−17.0 ± 111.9 (47)	−10.8 ± 95.7 (102)
LDL‐cholesterol, mg/dl			
Baseline, mean ± SD (*n*)	115.6 ± 32.3 (82)	117.9 ± 34.9 (104)	116.9 ± 33.7 (186)
Change at Week 56, mean ± SD (*n*)	4.0 ± 19.7 (55)	5.8 ± 33.0 (47)	4.8 ± 26.5 (102)
HDL‐cholesterol, mg/dl			
Baseline, mean ± SD (*n*)	60.9 ± 15.5 (82)	57.0 ± 16.3 (104)	58.8 ± 16.0 (186)
Change at Week 56, mean ± SD (*n*)	−1.0 ± 8.5 (55)	5.5 ± 10.3 (47)	2.0 ± 9.9 (102)
Glucose, mg/dl			
Baseline, mean ± SD (*n*)	92.2 ± 7.6 (43)	94.1 ± 9.4 (60)	93.3 ± 8.7 (103)
Change at Week 56, mean ± SD (*n*)	−0.7 ± 6.9 (31)	2.0 ± 9.2 (26)	0.5 ± 8.1 (57)
Prolactin, ng/ml			
Male			
Baseline, mean ± SD (*n*)	2.5 ± 3.4 (34)	25.1 ± 19.7 (53)	16.3 ± 19.1 (87)
Change at Week 56, mean ± SD (*n*)	5.3 ± 3.4 (30)	−22.8 ± 23.4 (28)	−8.3 ± 21.6 (58)
Female			
Baseline, mean ± SD (n)	8.0 ± 5.7 (48)	44.4 ± 54.7 (51)	26.8 ± 43.3 (99)
Change at Week 56, mean ± SD (*n*)	13.9 ± 7.4 (25)	−33.7 ± 66.4 (19)	−6.6 ± 49.4 (44)

Abbreviations: BMI, body mass index; HDL, high‐density lipoprotein; LDL, low‐density lipoprotein; SD, standard deviation.

With regard to body weight, both groups showed slight increases in mean (SD) body weight over 56 weeks of 0.4 (4.6) kg in the nonaripiprazole group and 1.1 (4.4) kg in the aripiprazole group (Table 3). Mean weight increase over 56 weeks was also seen in the risperidone/paliperidone group (0.4 [5.1] kg) but decreased slightly in the olanzapine/quetiapine group (−0.2 [4.1] kg, Table S6). Furthermore, a slightly lower proportion of patients in the nonaripiprazole group (18.8% [19/101]) experienced a body weight increase ≥7%, compared with the aripiprazole group (23.2% [19/82]). Conversely, a higher proportion of patients in the nonaripiprazole group (23.8% [24/101]) had a body weight decrease ≥7%, compared with the aripiprazole group (4.9% [4/82]).

**TABLE 3:** Changes in mean weight and proportion of patients with weight gain or loss over 56 weeks (observed cases)

	Aripiprazole			Non‐aripiprazole			Total		
Weight, kg									
Baseline, mean ± SD (n)	66.5 ± 13.7 (82)			64.7 ± 14.8 (104)			65.5 ± 14.3 (186)		
Change at week 28, mean ± SD (n)	1.3 ± 3.8 (60)			−0.1 ± 3.1 (55)			0.6 ± 3.5 (115)		
Change at Week 56, mean ± SD (n)	1.1 ± 4.4 (55)			0.4 ± 4.6 (47)			0.8 ± 4.5 (102)		

Weight gain or loss	N^a^	N^b^	%	N^a^	N^b^	%	N^a^	N^b^	%
Decrease ≥7%	82	4	4.9	101	24	23.8	183	28	15.3
Increase ≥7%	82	19	23.2	101	19	18.8	183	38	20.8
Baseline BMI <18.5 kg/m^2^	4	1	25.0	10	3	30.0	14	4	28.6
Baseline BMI ≥18.5 and < 25 kg/m^2^	39	8	20.5	49	9	18.4	88	17	19.3
Baseline BMI ≥25 and < 30 kg/m^2^	31	10	32.3	32	6	18.8	63	16	25.4
Baseline BMI ≥30 kg/m^2^	8	0	0.0	10	1	10.0	18	1	5.6

Abbreviations: BMI, body mass index; SD, standard deviation.

^a^ Number of safety subjects who had at least one post‐baseline numeric result for the given test.

^b^ Number of subjects with one post baseline potentially clinically relevant test abnormality observation.

The mean changes in body weight from baseline to Week 56 by BMI at baseline is shown in Figure S1. In both the aripiprazole group and nonaripiprazole group, mean (SD) increases in body weight were greater in patients with baseline BMI <18.5 kg/m^2^ (aripiprazole, 1.4 [2.0] kg; nonaripiprazole, 2.6 [5.4] kg) than in those with BMI ≥30 kg/m^2^ (aripiprazole, −2.7 [4.4] kg; nonaripiprazole, 1.8 [4.5] kg).

At baseline, prolactin levels were higher in the nonaripiprazole group than in the aripiprazole group. The mean change in prolactin levels was slightly increased in the aripiprazole group but decreased in the nonaripiprazole group in female and males (Table 2). Table S7 shows the change in prolactin levels by category (low, normal, and high). When switching from aripiprazole, the percentage of patients with low baseline prolactin values was high (male, 82.4% [28/34]; female, 45.8% [22/48]). At Week 56, many such patients with low baseline prolactin values shifted to normal values (male, 60.0% [15/25]; female, 100.0% [13/13]). When switching from nonaripiprazole, the percentage of patients with high baseline prolactin values was high (male, 71.7% [38/53]; female, 43.1% [22/51]). Similarly, at Week 56, many patients with high baseline prolactin values shifted to normal values (male, 72.7% [16/22]; female, 60.0% [6/10]).

### 3.3 Other safety assessment items

TEAEs occurred in 161 of 186 (86.6%) patients overall, 69 of 82 (84.1%) patients in the aripiprazole group, and 92 of 104 (88.5%) patients in the nonaripiprazole group. The most common TEAEs (≥10% of patients) in the aripiprazole group were nasopharyngitis (36.6%, *n* = 30), schizophrenia (15.9%, *n* = 13), headache (13.4%, *n* = 11), akathisia (12.2%, *n* = 10), somnolence (12.2%, *n* = 10) and diarrhea (11.0%, *n* = 9), whereas the most common TEAEs in the nonaripiprazole group were schizophrenia (29.8%, *n* = 31), nasopharyngitis, (26.9%, *n* = 28) and insomnia (13.5%, *n* = 14). Serious TEAE occurred in 9.8% (*n* = 8) and 14.4% (*n* = 15) in the aripiprazole and nonaripiprazole groups, respectively, with almost all serious events being the emergence of schizophrenia symptoms (aripiprazole, 8.5% [*n* = 7]; nonaripiprazole, 11.5% [*n* = 12]). There were no deaths recorded during the study.

Regarding extrapyramidal symptoms, there were few notable changes in any of the scales in either the aripiprazole or nonaripiprazole groups (Table S2). Although absolute decreases in AIMS and DIEPSS scores were higher in the nonaripiprazole group, the values at baseline were higher in the nonaripiprazole group and the final values at Week 56 were relatively similar between groups. Finally, there were no notable changes in QTc interval overall as well as in both the aripiprazole and nonaripiprazole groups (Table S3). The TEAE of QT prolongation was observed in one patient in both groups, respectively.

### 3.4 Efficacy

In terms of changes in psychiatric symptoms, mean changes in PANSS total and CGI‐S scores showed similar small decreases from baseline to Week 56 in both the aripiprazole and nonaripiprazole groups. Specifically, the mean (SD) change in PANSS total score was −8.4 (12.6) in the aripiprazole group (*n* = 55) and −12.1 (13.9) in the nonaripiprazole group (*n* = 47) with similar relative decreases from baseline levels in both groups. Changes in the PANSS positive, negative, and general psychopathology subscale scores showed comparable trends (−1.3 [2.8], −2.6 [3.9], −4.5 [7.4] in the aripiprazole group and −2.3 [4.0], −3.5 [4.9], −6.3 [7.5] in the nonaripiprazole group, respectively). Similarly, the mean (SD) change in CGI‐S score was −0.3 (0.7) in the aripiprazole group (*n* = 55) and −0.4 (0.7) in the nonaripiprazole group (*n* = 47).

## 4 DISCUSSION

In this study, we investigated the long‐term safety of patients with schizophrenia who switched to brexpiprazole. There were some differences in the baseline characteristics of aripiprazole and nonaripiprazole patients, with aripiprazole patients, on average, being younger by 9.0 years and having a shorter duration of illness by 8.3 years. In addition, aripiprazole‐treated patients had less severe psychiatric symptoms by a mean of 10 points for PANSS and 0.5 points for CGI‐S as well as receiving a lower mean dose of antipsychotic treatment. Accordingly, it appears that the aripiprazole group was generally younger and might have had less severe or prolonged disease.

The discontinuation rate was numerically lower in the group that received aripiprazole as a pretreatment antipsychotic (32.9%) than in the group that received a nonaripiprazole antipsychotic (54.8%). Adverse events have been reported to be associated with discontinuation of treatment (Essock et al., 2006). In this study, aripiprazole had a lower rate of adverse events leading to discontinuation (aripiprazole, 8.5%; nonaripiprazole, 15.4%), which may have affected the rate of discontinuation. In order to reduce discontinuation, it is important to provide treatment that does not cause adverse events. Further, gradual switching of antipsychotic drugs may reduce adverse events and subsequent discontinuation (Newcomer et al., 2013). Therefore, the switching period used in this study (4 weeks) may need to be extended.

The propensity toward various adverse events, such as metabolic abnormalities, sexual dysfunction, and extrapyramidal symptoms, differs between individual antipsychotic agents and is hence considered a key consideration when switching antipsychotic medication, especially for reasons related to tolerability and safety (Buckley & Correll, 2008). In this analysis, switching to brexpiprazole for 56 weeks was not associated with appreciable changes in extrapyramidal symptoms, QTc interval, levels of cholesterol, LDL‐cholesterol, HDL‐cholesterol, or blood glucose. However, it should be noted that this may reflect the fact that many patients enrolled in this trial had few abnormalities at baseline.

Schizophrenia is associated with a poor prognosis and increased mortality, especially in relation to cardiovascular mortality (Hennekens et al., 2005; Nielsen, Uggerby, Jensen, & McGrath, 2013). Therefore, it is important to provide antipsychotic treatment with a low propensity to cause weight gain. Switching to brexpiprazole appears to have a low impact on weight gain in patients with schizophrenia as reported in a number of previous studies (Huhn et al., 2019; McEvoy & Citrome, 2016; Taylor et al., 2018). In one comprehensive meta‐analysis, only small differences compared with placebo were noted in mean body weight for aripiprazole (0.48 kg) and brexpiprazole (0.70 kg) whereas large differences were noted among the non‐aripiprazole group, including olanzapine (2.78 kg), quetiapine (1.94 kg), risperidone (1.44 kg), and paliperidone (1.49 kg; Huhn et al., 2019). Furthermore, a randomized, comparative trial found that risperidone, quetiapine, and olanzapine led to mean weight changes of 3.6–4.6 kg over 24 weeks (Newcomer et al., 2009) which is greater than mean changes at Week 28 for brexpiprazole patients previously treated with aripiprazole (1.3 kg) and non‐aripiprazole (−0.1 kg) in the present analysis and consistent with the low risk of weight gain with brexpiprazole noted elsewhere (Huhn et al., 2019; McEvoy & Citrome, 2016; Taylor et al., 2018). As patients in the present analysis previously treated with non‐aripiprazole antipsychotics had less weight gain and a higher proportion of weight loss, it seems more likely that weight loss can be expected if the previous treatment involves a non‐aripiprazole antipsychotic given the lower risk of weight gain with aripiprazole than with other atypical antipsychotics noted elsewhere (Cooper et al., 2016; Huhn et al., 2019; McEvoy & Citrome, 2016; Taylor et al., 2018).

Antipsychotics are known to produce hyperprolactinemia in up to about 70% of patients with schizophrenia, which can lead to sexual dysfunction, gynecomastia in men, and menstrual abnormalities in women, and osteoporosis in the long‐term (de Boer et al., 2015; Galletly et al., 2016; Hasan et al., 2013; Inder & Castle, 2011; Worsley, Santoro, Miller, Parish, & Davis, 2016). In the present analysis, mean change of prolactin levels were slightly elevated following brexpiprazole treatment in the aripiprazole group (male, 5.3 ng/ml; female, 13.9 ng/ml), but the prolactin levels were within the normal range. On the other hand, switching to brexpiprazole decreased mean prolactin levels in the non‐aripiprazole group (male, −22.8 ng/ml; female, −33.7 ng/ml). In the nonaripiprazole group, most patients with hyperprolactinemia underwent normalization of their prolactin levels. This result is consistent with those from a large‐scale meta‐analysis, which found that brexpiprazole had a neutral effect on prolactin levels compared with aripiprazole, which was associated with low prolactin levels, and some non‐aripiprazole antipsychotics (risperidone and paliperidone), which tended to increase prolactin levels (Huhn et al., 2019). As sexual dysfunction due to hyperprolactinemia adversely affects adherence and quality of life in schizophrenia (Cutler, 2003; Olfson et al., 2005) antipsychotic switching may lead to improvements in adherence and quality of life as well as sexual dysfunction. Various schizophrenia guidelines have also reported hyperprolactinemia to be a risk factor for osteoporosis (Galletly et al., 2016; Hasan et al., 2013; Taylor et al., 2018) which may also be mitigated by switching to brexpiprazole (Halbreich, Kinon, Gilmore, & Kahn, 2003).

In the present study, a slight improvement in psychiatric symptoms based on PANSS and CGI‐S was observed in both aripiprazole and non‐aripiprazole groups. It is possible that relatively stable patients such as those in this study are at low risk of worsening psychotic symptoms due to switching.

The present post‐hoc analysis is associated with several limitations. First, it is difficult to generalize these results to real‐world settings given that enrolled patients were required to meet inclusion and exclusion criteria. Second, there are no data allowing comparison to strategies based on switching to other antipsychotics. Finally, patient numbers in some analyzed groups (especially those in relation to body weight) were low, which also reduces the confidence and generalizability surrounding these results. Despite these limitations, a key strength of this analysis is that it was drawn from a high‐quality data set.

In conclusion, the present demonstrated that switching to brexpiprazole was associated with a low risk for metabolic abnormalities (including weight gain), hyperprolactinemia, extrapyramidal symptoms and changes in QTc interval in the long‐term treatment. In addition, brexpiprazole was associated with minimal change in psychiatric symptoms. Brexpiprazole may be considered as a treatment option if there are safety or tolerability issues, especially those related to metabolic abnormalities or hyperprolactinemia, with current antipsychotic therapy.

## CONFLICTS OF INTEREST

Jun Ishigooka has received research support or speakers' honoraria from, or has served as a consultant to Novartis, Otsuka Pharmaceutical Co., Ltd, Sumitomo Dainippon Pharma, Eli Lilly Japan, and Takeda Pharmaceutical Co., Ltd. Ken Inada has received speaker's honoraria from Eisai, Eli Lilly Japan, Janssen, Meiji Seika Pharma, Mitsubishi Tanabe Pharma, Mochida, MSD, Novartis, Otsuka Pharmaceutical Co., Ltd., Shionogi, Sumitomo Dainippon Pharma, and Yoshitomiyakuhin Corporation, and research grants from the Japan Agency for Medical Research and Development (AMED), Ministry of Health, Labor and Welfare, Mitsubishi Tanabe Pharma, MSD, and National Center of Neurology and Psychiatry (NCNP). Kazunari Niidome, Kazuo Aoki, Yoshitsugu Kojima, Shuichi Iwashita and Sakiko Yamada are employees of Otsuka Pharmaceutical Co., Ltd.

## AUTHOR CONTRIBUTIONS

Jun Ishigooka advised on the study design and interpretation of the data. Ken Inada contributed to the conception and interpretation of the study. Shuichi Iwashita designed the study, wrote the protocol, supervised the data acquisition and monitoring activity, and contributed to interpretation of the data. Kazunari Niidome wrote the outline of the manuscript. Kazuo Aoki, Yoshitsugu Kojima, and Sakiko Yamada contributed to the study design and interpretation. All authors contributed to and approved the final manuscript.

## Supporting information

Supplementary Material

## Data Availability

The data that support the findings of this study are proprietary in nature and are not publicly available. However, the data are available on request from the corresponding author upon reasonable request.
