# Supplementary material for: Safety of switching to brexpiprazole in Japanese patients with schizophrenia: A post‐hoc analysis of a long‐term open‐label study
Source: Hum Psychopharmacol. 2021 Jan 26;36(4):e2777. doi: 10.1002/hup.2777 (PMC8365679; doi:10.1002/hup.2777)
Supplement: Supplementary file 1 — Supplementary Material [file HUP-36-e2777-s001.docx]

# Supplementary Material

**Supplementary Table 1.** Major exclusion criteria and prohibited medications

| **Major exclusion criteria**   - First-episode schizophrenia - Severe akathisia - Clinically significant tardive dyskinesia - DSM-IV-TR diagnostic criteria other than schizophrenia - History of substance abuse or dependence within the past 180 days - Electroconvulsive treatment 60 days prior to informed consent. - Complications or history of diabetes - Judged by the investigators to be inappropriate for inclusion in this trial |
| --- |
| **Prohibited medications**   - All psychotropic agents, including antipsychotics (except during switching phase), antidepressants, mood stabilizers, and benzodiazepines (except for as short-term rescue therapy for the treatment of agitation and insomnia), - Ramelteon, suvorexant, nonbenzodiazepine sleep aids (except for limited treatment of insomnia) - Antihistamines (excluding medicine for treatment of a complication other than a psychiatric disorder and external application) - Beta-blockers (except when used for the treatment of cardiovascular disease) - Varenicline, and supplements containing centrally acting substances (tryptophan and St. John’s wort) |

**Supplementary Table 2.** Changes in extrapyramidal symptom scales (observed cases)

|  | **Aripiprazole** | **Non-aripiprazole** | **Total** |
| --- | --- | --- | --- |
| AIMS |  |  |  |
| Baseline, mean ± SD (n) | 0.2 ± 1.0 (82) | 0.7 ± 1.7 (104) | 0.5 ± 1.4 (186) |
| Change at Week 56, mean ± SD (n) | 0.0 ± 0.4 (55) | -0.4 ± 1.3 (47) | -0.2 ± 0.9 (102) |
| BARS |  |  |  |
| Baseline, mean ± SD (n) | 0.1 ± 0.4 (82) | 0.2 ± 0.6 (104) | 0.2 ± 0.5 (186) |
| Change at Week 56, mean ± SD (n) | -0.1 ± 0.4 (55) | -0.1 ± 0.4 (47) | -0.1 ± 0.4 (102) |
| DIEPSS |  |  |  |
| Baseline, mean ± SD (n) | 0.8 ± 1.9 (82) | 1.9 ± 3.2 (104) | 1.4 ± 2.7 (186) |
| Change at Week 56, mean ± SD (n) | -0.1 ± 1.0 (55) | -0.8 ± 2.4 (47) | -0.4 ± 1.8 (102) |

Abbreviations: AIMS, Abnormal Involuntary Movement Scale (AIMS); BARS, Barns Akathisia Rating Scale (BARS); DIEPSS, Drug-induced Extrapyramidal Symptoms Scale (DIEPSS); SD, standard deviation

**Supplementary Table 3.** Changes in QTc interval (observed cases)

|  | **Aripiprazole** | **Non-aripiprazole** | **Total** |
| --- | --- | --- | --- |
| QTc interval, Bazett, msec |  |  |  |
| Baseline, mean ± SD (n) | 415.4 ± 21.7 (82) | 420.7 ± 20.4 (104) | 418.4 ± 21.1 (186) |
| Change at Week 56, mean ± SD (n) | 0.5 ± 17.1 (56) | -1.2 ± 21.8 (47) | -0.3 ± 19.3 (103) |
| QTc interval, Fridericia, msec |  |  |  |
| Baseline, mean ± SD (n) | 408.2 ± 17.5 (82) | 409.2 ± 17.0 (104) | 408.8 ± 17.2 (186) |
| Change at Week 56, mean ± SD (n) | 3.8 ± 13.6 (56) | 4.2 ± 16.7 (47) | 4.0 ± 15.0 (103) |
| QTcN interval, msec |  |  |  |
| Baseline, mean ± SD (n) | 409.7 ± 17.6 (82) | 411.7 ± 16.9 (104) | 410.8 ± 17.2 (186) |
| Change at Week 56, mean ± SD (n) | 3.2 ± 13.8 (56) | 3.1 ± 17.2 (47) | 3.2 ± 15.3 (103) |

Abbreviations: QTc, corrected QT interval; QTcN, population-specific QT correction; SD, standard deviation

**Supplementary Table 4.** Demographic and baseline clinical characteristics (observed cases)

|  | **Aripiprazole (n = 82)** | **RIS+PPD**  **(n = 49)** | **OLZ+QTP**  **(n = 44)** | **Total^a^ (n =175 )** |
| --- | --- | --- | --- | --- |
| Age (years), mean ± SD | 40.1 ± 12.9 | 48.2 ± 13.6 | 51.1 ± 16.8 | 45.1 ± 14.9 |
| Duration of disease (years), mean ± SD | 12.1 ± 10.6 | 20.9 ± 13.8 | 22.1 ± 16.9 | 17.1 ± 14.1 |
| Female sex, n (%) | 48 (58.5) | 24 (49.0) | 21 (47.7) | 93 (53.1) |
| Body weight (kg), mean ± SD | 66.5 ± 13.7 | 67.2 ± 15.7 | 62.3 ± 13.9 | 65.6 ± 14.4 |
| BMI (kg/m^2^), mean ± SD | 24.9 ± 4.1 | 25.2 ± 4.7 | 23.8 ± 4.7 | 24.7 ± 4.4 |
| <18.5, n (%) | 4 (4.9) | 5 (10.2) | 4 (9.1) | 13 (7.4) |
| 18.5 to < 25, n (%) | 39 (47.6) | 22 (44.9) | 22 (50.0) | 83 (47.4) |
| 25 to < 30, n (%) | 31 (37.8) | 14 (28.6) | 16 (36.4) | 61 (34.9) |
| ≥30, n (%) | 8 (9.8) | 8 (16.3) | 2 (4.5) | 18 (10.3) |
| CP equivalent dose of all antipsychotics (mg/day), mean ± SD | 433.4 ± 258.8 | 590.1 ± 462.5 | 593.8 ± 384.0.4 | 517.6 ± 364.6 |
| Number of pretreatment antipsychotic drugs, n (%) | | | | |
| 1 drug | 72 (87.8) | 28 (57.1) | 35 (79.5) | 135 (77.1) |
| 2 drugs | 9 (11.0) | 16 (32.7) | 6 (13.6) | 31 (17.7) |
| ≥3 drugs | 1 (1.2) | 5 (10.2) | 3 (6.8) | 9 (5.1) |
| PANSS total score, mean ± SD | 64.6 ± 19.1 | 72.7 ± 23.2 | 78.6 ± 23.3 | 70.4 ± 22.0 |
| CGI-S, mean ± SD | 3.2 ± 0.9 | 3.7 ± 1.2 | 3.7 ± 1.2 | 3.4 ± 1.1 |
| Concomitant medication use |  |  |  |  |
| Benzodiazepines, n (%) | 33 (40.2) | 31 (63.3) | 18 (40.9) | 82 (46.9) |
| Non-benzodiazepines, n (%) | 1 (1.2) | 0 (0.0) | 1 (2.3) | 2 (1.1) |
| Antiparkinsonian drugs, n (%) | 8 (9.8) | 14 (28.6) | 6 (13.6) | 28 (16.0) |
| Laxatives, n (%) | 9 (11.0) | 12 (24.5) | 16 (36.4) | 37 (21.1) |

Abbreviations: BMI, body mass index; CP, chlorpromazine; CGI-S, Clinical Global Impression-Severity; OLZ, olanzapine; PANSS, Positive and Negative Symptom Severity; PPD, paliperidone; QTP, quetiapine; RIS, risperidone; SD, standard deviation.

^a^aripiprazole, RIS, PPD, OLZ, QTP

RIS+PPD breakdown: risperidone (n=33), paliperidone (n=16); OLZ+QTP breakdown: olanzapine (n=36), quetiapine (n=8)

**Supplementary Table 5.** Changes in lipid, blood glucose and prolactin levels during 56-week brexpiprazole treatment in patients previously treated with aripiprazole and non-aripiprazole antipsychotics (observed cases)

|  | **Aripiprazole** | **RIS+PPD** | **OLZ+QTP** | **Total^a^** |
| --- | --- | --- | --- | --- |
| Total cholesterol, mg/dL |  |  |  |  |
| Baseline, mean ± SD (n) | 191.7 ± 36.7 (82) | 186.6 ± 39.1 (49) | 195.2 ± 38.4 (44) | 191.7 ± 37.7 (175) |
| Change at Week 56, mean ± SD (n) | 3.4 ± 23.9 (55) | 15.0 ± 23.8 (22) | 4.8 ± 44.8 (20) | 6.3 ± 29.5 (97) |
| Triglycerides, mg/dL | | | | |
| Baseline, mean ± SD (n) | 120.8 ± 111.5 (82) | 117.1 ± 89.0 (49) | 129.3 ± 104.7 (44) | 121.9 ± 103.4 (175) |
| Change at Week 56, mean ± SD (n) | -5.4 ± 79.9 (55) | -12.6 ± 56.7 (22) | -16.0 ± 111.1 (20) | -9.2 ± 82.2 (97) |
| LDL-cholesterol, mg/dL | | | | |
| Baseline, mean ± SD (n) | 115.6 ± 32.3 (82) | 116.1 ± 36.3 (49) | 121.2 ± 36.9 (44) | 117.1 ± 34.5 (175) |
| Change at Week 56, mean ± SD (n) | 4.0 ± 19.7 (55) | 11.2 ± 26.7 (22) | 0.2 ± 39.4 (20) | 4.8 ± 26.4 (97) |
| HDL-cholesterol, mg/dL | | | | |
| Baseline, mean ± SD (n) | 60.9 ± 15.5 (82) | 57.4 ± 17.0 (49) | 56.1 ± 15.9 (44) | 58.7 ± 16.1 (175) |
| Change at Week 56, mean ± SD (n) | -1.0 ± 8.5 (55) | 5.5 ± 10.4 (22) | 7.2 ± 10.0 (20) | 2.2 ± 9.9 (97) |
| Glucose, mg/dL | | | | |
| Baseline, mean ± SD (n) | 92.2 ± 7.6 (43) | 97.0 ± 10.8 (26) | 92.2 ± 8.1 (26) | 93.5 ± 8.9 (95) |
| Change at Week 56, mean ± SD (n) | -0.7 ± 6.9 (31) | 0.3 ± 9.1 (11) | 0.8 ± 7.0 (12) | -0.2 ± 7.3 (54) |
| Prolactin, ng/mL |  |  |  |  |
| Male |  |  |  |  |
| Baseline, mean ± SD (n) | 2.5 ± 3.4 (34) | 28.7 ± 17.7 (25) | 23.0 ± 22.9 (23) | 16.2 ± 19.5 (82) |
| Change at Week 56, mean ± SD (n) | 5.3 ± 3.4 (30) | -26.0 ± 20.0 (14) | -22.5 ± 26.8 (12) | -8.5 ± 21.7 (56) |
| Female |  |  |  |  |
| Baseline, mean ± SD (n) | 8.0 ± 5.7 (48) | 67.6 ± 69.6 (24) | 22.4 ± 17.3 (21) | 26.6 ± 43.8 (93) |
| Change at Week 56, mean ± SD (n) | 13.9 ± 7.4 (25) | -76.6 ± 85.9 (8) | -4.2 ± 16.7 (8) | -7.3 ± 51.1 (41) |

Abbreviations: BMI, body mass index; HDL, high-density lipoprotein; LDL, low-density lipoprotein; OLZ, olanzapine; PPD, paliperidone; QTP, quetiapine RIS, risperidone; SD, standard deviation.

^a^aripiprazole, RIS, PPD, OLZ, QTP

**Supplementary Table 6.** Changes in mean weight and proportion of patients with weight gain or loss over 56 weeks (observed cases)

|  | **Aripiprazole** | | | **RIS+PPE** | | | **OLZ+QTP** | | | **Total^c^** | | |
| --- | --- | --- | --- | --- | --- | --- | --- | --- | --- | --- | --- | --- |
| Weight, kg |  | | |  | | |  | | |  | | |
| Baseline, mean ± SD (n) | 66.5 ± 13.7 (82) | | | 67.2 ± 15.7 (49) | | | 62.3 ± 13.9 (44) | | | 65.6 ± 14.4 (175) | | |
| Change at week 28, mean ± SD (n) | 1.3 ± 3.8 (60) | | | 0.3 ± 3.5 (27) | | | -1.3 ± 1.8 (21) | | | 0.5 ± 3.5 (108) | | |
| Change at Week 56, mean ± SD (n) | 1.1 ± 4.4 (55) | | | 0.4 ± 5.1 (22) | | | -0.2 ± 4.1 (20) | | | 0.7 ± 4.5 (97) | | |
| Weight gain or loss | N^a^ | N^b^ | % | N^a^ | N^b^ | % | N^a^ | N^b^ | % | N^a^ | N^b^ | % |
| Decrease ≥7% | 82 | 4 | 4.9 | 47 | 10 | 21.3 | 43 | 12 | 27.9 | 172 | 26 | 15.1 |
| Increase ≥7% | 82 | 19 | 23.2 | 47 | 10 | 21.3 | 43 | 4 | 9.3 | 172 | 33 | 19.2 |
| Baseline BMI <18.5 kg/m^2^ | 4 | 1 | 25.0 | 5 | 1 | 20.0 | 4 | 1 | 25.0 | 13 | 3 | 23.1 |
| Baseline BMI ≥18.5 and <25 kg/m^2^ | 39 | 8 | 20.5 | 21 | 4 | 19.0 | 22 | 2 | 9.1 | 82 | 14 | 17.1 |
| Baseline BMI ≥25 and <30 kg/m^2^ | 31 | 10 | 32.3 | 14 | 4 | 28.6 | 15 | 1 | 6.7 | 60 | 15 | 25.0 |
| Baseline BMI ≥30 kg/m^2^ | 8 | 0 | 0.0 | 7 | 1 | 14.3 | 2 | 0 | 0.0 | 17 | 1 | 5.9 |

Abbreviations: BMI, body mass index; OLZ, olanzapine; PPD, paliperidone; QTP, quetiapine; RIS, risperidone; SD, standard deviation.

^a^Number of safety subjects who had at least one post-baseline numeric result for the given test; ^b^Number of subjects with one post baseline potentially clinically relevant test abnormality observation; ^c^aripiprazole, RIS, PPD, OLZ, QTP.

**Supplementary Table 7.** Shifts in prolactin Status in males and females previously treated with (A) aripiprazole and (B) non-aripiprazole antipsychotics (observed cases)

| **Male** | **Baseline** | |  | **Week 4** | | |  | **Week 8** | | |  | **Week 12** | | |  | **Week 28** | | |  | **Week 56** | | | |
| --- | --- | --- | --- | --- | --- | --- | --- | --- | --- | --- | --- | --- | --- | --- | --- | --- | --- | --- | --- | --- | --- | --- | --- |
| **Pretreatment antipsychotics drug** | **category** | **n (%)** |  | **L n (%)** | **N n (%)** | **H n (%)** |  | **L n (%)** | **N n (%)** | **H n (%)** |  | **L n (%)** | **N n (%)** | **H n (%)** |  | **L n (%)** | **N n (%)** | **H n (%)** |  | **L n (%)** | **N n (%)** | **H n (%)** |  |
| aripiprazole | L | 28 (82.4) |  | 23 (82.1) | 5 (17.9) | - |  | 12 (42.9) | 16 (57.1) | - |  | 11 (39.3) | 17 (60.7) | - |  | 9 (34.6) | 16 (61.5) | 1 (3.8) |  | 9 (36.0) | 15 (60.0) | 1 (4.0) |  |
|  | N | 5 (14.7) |  | - | 4 (80.0) | 1 (20.0) |  | - | 2 (40.0) | 3 (60.0) |  | - | 2 (40.0) | 3 (60.0) |  | - | 2 (50.0) | 2 (50.0) |  | - | 2 (50.0) | 2 (50.0) |  |
|  | H | 1 (2.9) |  | - | - | 1 (100.0) |  | - | - | 1 (100.0) |  | - | - | 1 (100.0) |  | - | - | 1 (100.0) |  | - | - | 1 (100.0) |  |
| non-aripiprazole | L | - |  | - | - | - |  | - | - | - |  | - | - | - |  | - | - | - |  | - | - | - |  |
|  | N | 15 (28.3) |  | 3 (23.1) | 7 (53.8) | 3 (23.1) |  | 2 (22.2) | 6 (66.7) | 1 (11.1) |  | 1 (12.5) | 5 (62.5) | 2 (25.0) |  | 2 (28.6) | 3 (42.9) | 2 (28.6) |  | 2 (33.3) | 2 (33.3) | 2 (33.3) |  |
|  | H | 38 (71.7) |  | 1 (2.7) | 20 (54.1) | 16 (43.2) |  | 3 (8.8) | 23 (67.6) | 8 (23.5) |  | 3 (9.1) | 22 (66.7) | 8 (24.2) |  | 5 (20.8) | 16 (66.7) | 3 (12.5) |  | 4 (18.2) | 16 (72.7) | 2 (9.1) |  |
| Total^a^ | L | 28 (32.2) |  | 23 (82.1) | 5 (17.9) | - |  | 12 (42.9) | 16 (57.1) | - |  | 11 (39.3) | 17 (60.7) | - |  | 9 (34.6) | 16 (61.5) | 1 (3.8) |  | 9 (36.0) | 15 (60.0) | 1 (4.0) |  |
|  | N | 20 (23.0) |  | 3 (16.7) | 11 (61.1) | 4 (22.2) |  | 2 (14.3) | 8 (57.1) | 4 (28.6) |  | 1 (7.7) | 7 (53.8) | 5 (38.5) |  | 2 (18.2) | 5 (45.5) | 4 (36.4) |  | 2 (20.0) | 4 (40.0) | 4 (40.0) |  |
|  | H | 39 (44.8) |  | 1 (2.6) | 20 (52.6) | 17 (44.7) |  | 3 (8.6) | 23 (65.7) | 9 (25.7) |  | 3 (8.8) | 22 (64.7) | 9 (26.5) |  | 5 (20.0) | 16 (64.0) | 4 (16.0) |  | 4 (17.4) | 16 (69.6) | 3 (13.0) |  |
|  |  |  |  |  |  |  |  |  |  |  |  |  |  |  |  |  |  |  |  |  |  |  |  |
| **Female** | **Baseline** | |  | **Week 4** | | |  | **Week 8** | | |  | **Week 12** | | |  | **Week 28** | | |  | **Week 56** | | | |
| **Pretreatment antipsychotics drug** | **category** | **n (%)** |  | **L n (%)** | **N n (%)** | **H n (%)** |  | **L n (%)** | **N n (%)** | **H n (%)** |  | **L n (%)** | **N n (%)** | **H n (%)** |  | **L n (%)** | **N n (%)** | **H n (%)** |  | **L n (%)** | **N n (%)** | **H n (%)** |  |
| aripiprazole | L | 22 (45.8) |  | 10 (47.6) | 11 (52.4) | - |  | 1 (4.8) | 19 (90.5) | 1 (4.8) |  | - | 19 (90.5) | 2 (9.5) |  | 1 (6.7) | 13 (86.7) | 1 (6.7) |  | - | 13 (100.0) | - |  |
|  | N | 26 (54.2) |  | - | 24 (96.0) | 1 (4.0) |  | - | 17 (73.9) | 6 (26.1) |  | - | 19 (82.6) | 4 (17.4) |  | - | 9 (60.0) | 6 (40.0) |  | - | 9 (75.0) | 3 (25.0) |  |
|  | H | - |  | - | - | - |  | - | - | - |  | - | - | - |  | - | - | - |  | - | - | - |  |
| non-aripiprazole | L | 2 (3.9) |  | - | 2 (100.0) | - |  | - | 2 (100.0) | - |  | - | 2 (100.0) | - |  | - | 2 (100.0) | - |  | - | 2 (100.0) | - |  |
|  | N | 27 (52.9) |  | 2 (8.0) | 16 (64.0) | 7 (28.0) |  | 1 (5.0) | 16 (80.0) | 3 (15.0) |  | 1 (5.6) | 15 (83.3) | 2 (11.1) |  | 1 (9.1) | 9 (81.8) | 1 (9.1) |  | 1 (14.3) | 6 (85.7) | - |  |
|  | H | 22 (43.1) |  | 1 (5.6) | 9 (50.0) | 8 (44.4) |  | 2 (13.3) | 9 (60.0) | 4 (26.7) |  | - | 11 (73.3) | 4 (26.7) |  | - | 9 (75.0) | 3 (25.0) |  | 1 (10.0) | 6 (60.0) | 3 (30.0) |  |
| Total^a^ | L | 24 (24.2) |  | 10 (43.5) | 13 (56.5) | - |  | 1 (4.3) | 21 (91.3) | 1 (4.3) |  | - | 21 (91.3) | 2 (8.7) |  | 1 (5.9) | 15 (88.2) | 1 (5.9) |  | - | 15 (100.0) | - |  |
|  | N | 53 (53.5) |  | 2 (4.0) | 40 (80.0) | 8 (16.0) |  | 1 (2.3) | 33 (76.7) | 9 (20.9) |  | 1 (2.4) | 34 (82.9) | 6 (14.6) |  | 1 (3.8) | 18 (69.2) | 7 (26.9) |  | 1 (5.3) | 15 (78.9) | 3 (15.8) |  |
|  | H | 22 (22.2) |  | 1 (5.6) | 9 (50.0) | 8 (44.4) |  | 2 (13.3) | 9 (60.0) | 4 (26.7) |  | - | 11 (73.3) | 4 (26.7) |  | - | 9 (75.0) | 3 (25.0) |  | 1 (10.0) | 6 (60.0) | 3 (30.0) |  |

Abbreviations: APZ, aripiprazole; OLZ, olanzapine; PPD, paliperidone; QTP, quetiapine; RIS, risperidone.

Note: L: Low; N: Normal; H: High (as compared with normal range values)

^a^APZ, RIS, PPD, OLZ, QTP

Normal values: male 3.58-12.78 ng/mL, female 6.12-30.54 ng/mL

**Supplementary Figure 1.** Change in body weight from baseline to Week 56 according to baseline BMI (observed cases)


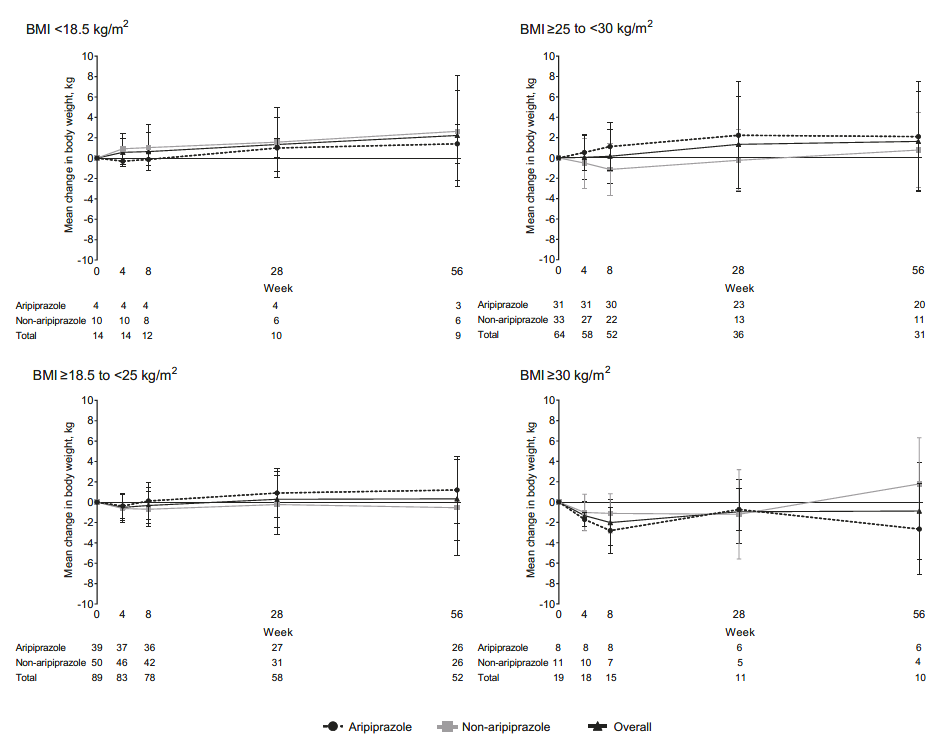


BMI, body mass index
